# Autoimmune Heparin-Induced Thrombocytopenia: A Diagnostic and Management Challenge After Transcatheter Aortic Valve Replacement

**DOI:** 10.7759/cureus.45453

**Published:** 2023-09-18

**Authors:** Aisha Batool, Shahzad Chaudhry, Ayesha Javaid, Ashley Kenney

**Affiliations:** 1 Internal Medicine, Columbia St. Mary Hospital, Milwaukee, USA; 2 Family Medicine, Advocate Aurora Healthcare, Milwaukee, USA; 3 Cardiology, Russells Hall Hospital, Dudley, GBR; 4 Hospital Medicine, Health Partners, Minneapolis, USA

**Keywords:** intravenous immunoglobulins (ivig), stroke, autoimmune thrombocytopenia, drug-induced thrombocytopenia, tavr (transcatheter aortic valve replacement)

## Abstract

Heparin-induced thrombocytopenia (HIT) is a commonly encountered condition, especially in inpatient settings, and is often attributed to high mortality and prolonged hospital stays. A rare entity, autoimmune heparin-induced thrombocytopenia (aHIT) refers to a condition in which antiplatelet factor-4 (PF4) antibodies activate platelets even in the absence of heparin. Our patient presented 12 days after transcatheter aortic valve replacement (TAVR) with altered mental status and severe thrombocytopenia. Further work-up revealed acute thromboembolic cerebrovascular accident (CVA),* *and the HIT antibody was positive. He was started on intravenous argatroban infusion with poor response. Platelet factor-4 antibodies were positive as well, and he was started on intravenous immunoglobulins (IVIG) therapy resulting in platelet recovery. This case is a reminder to consider autoimmune HIT, especially when platelet count fails to improve with conventional therapy.

## Introduction

The basis of heparin-induced thrombocytopenia (HIT) is an antibody that recognizes an epitope on the platelet factor-4 (PF4)-heparin complex. The antibody-PF4-heparin complex then binds to FcγRII receptors on the platelet surface and cross-links the receptors. This cross-linking results in aggressive platelet activation and aggregation, which further results in the activation of the blood-coagulation cascade. Hence thrombocytopenia results in a paradoxical hypercoagulable state, which further manifests as venous thromboembolism (VTE) [1]. Autoimmune heparin-induced thrombocytopenia (aHIT) causes a severe hypercoagulable state triggering a massive thrombin storm needing additional therapies and aggressive anticoagulation, apart from stopping heparin. Thrombocytopenia in these cases seems to be very severe and prolonged compared to classic HIT and poses additional clinical challenges in terms of anticoagulation management.

## Case presentation

We hereby present a unique case of a 79-year-old Caucasian male with a medical history of chronic persistent atrial fibrillation on chronic warfarin therapy, essential hypertension, severe aortic stenosis, chronic kidney disease stage 3a, congestive heart failure with reduced ejection fraction 35-40% and diabetes mellitus type 2 with peripheral neuropathy. The patient underwent elective transcatheter aortic valve replacement (TAVR) and was discharged home in stable condition. The patient returned to the emergency room 12 days after TAVR with confusion and generalized weakness. On physical examination he was found to be altered, with irregularly irregular heart rate of 96 beats per minute, respiratory rate of 18 breaths per minute, blood pressure of 133/57 mmHg, speech was slurred, lungs clear to auscultation, and spontaneous movement of all four extremities was noted, however, he was not following commands. 

**Figure 1:**
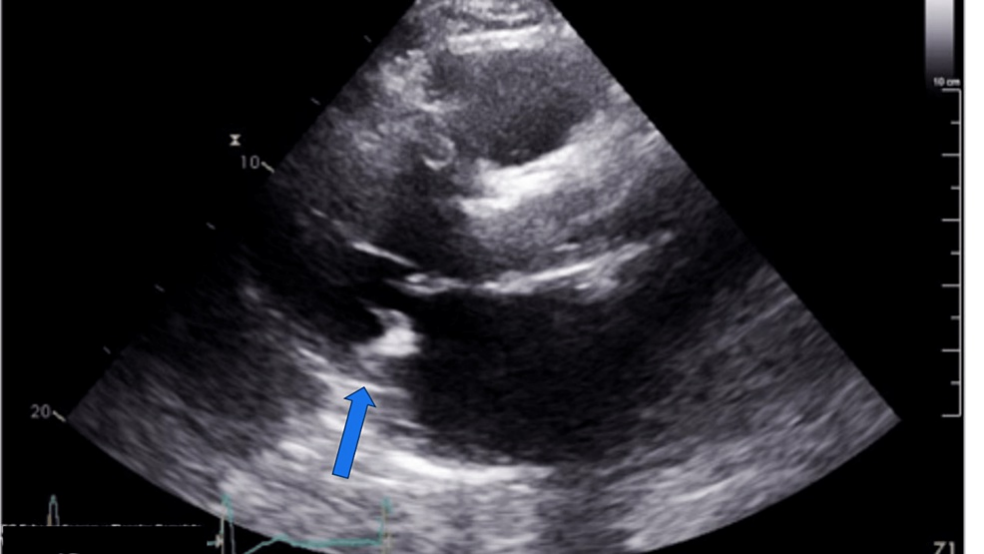
Parasternal long-axis view on TTE showing TAVR (blue arrow), no vegetation, and no para-valvular leak (not shown in the still image). TTE: trans-thoracic echocardiogram; TAVR: transcatheter aortic valve replacement.

Laboratory results revealed severe thrombocytopenia with platelet count 20x109/L, hemoglobin 11.5 g/dl, international normalized ratio (INR) 2.3, platelet count pre-operatively was 206×109/L, which 4 days after TAVR was 103×109/L. The patient received an intravenous unfractionated heparin infusion during his TAVR procedure 12 days ago. The CT scan of the brain was unremarkable. A limited transthoracic echocardiogram was done to evaluate TAVR and it showed no para-valvular or transvalvular leak and absence of vegetation (Figure 1).

**Figure 2:**
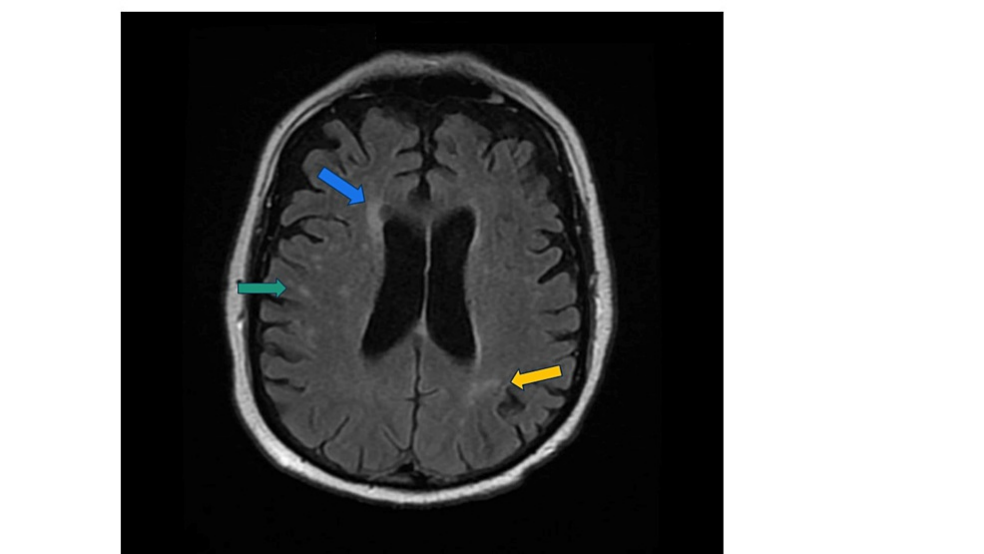
MRI brain without contrast showing bilateral (notably right>left), periventricular (blue arrow), parietal (green arrow), temporal (not shown here), and occipital (yellow arrow) lobe hypo-densities, representative of thromboembolic cerebrovascular accident.

MRI Brain without contrast revealed bilateral embolic ischemic CVA (Figure 2).

**Figure 3:**
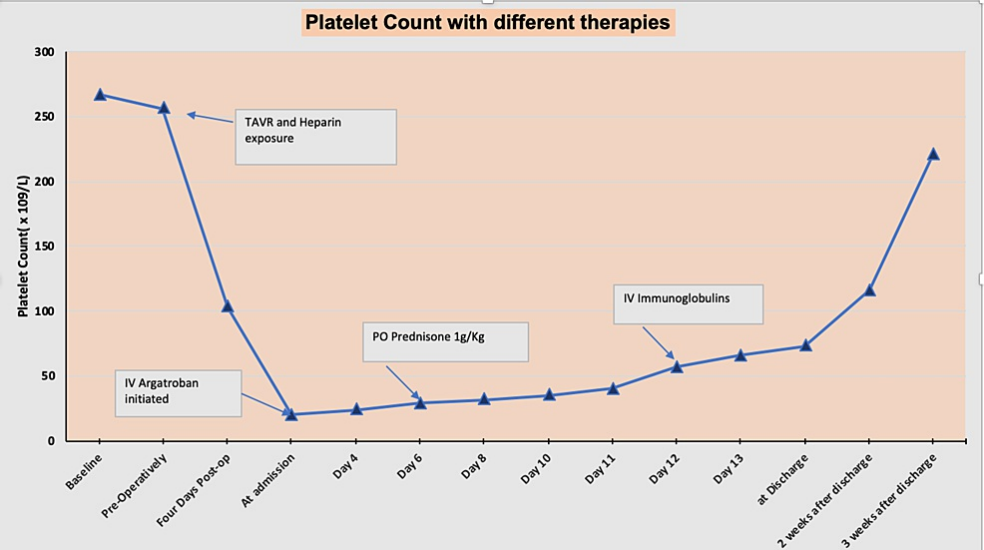
Graphical representation of the timeline of platelet count with different therapeutic options. IV: intravenous; PO: per oral; TAVR: transcatheter aortic valve replacement.

The patient was out of the alteplase (tPA) window and was also taking warfarin 3 mg at home. Due to a drop in his platelet count with recent exposure to heparin infusion, HIT antibody test and 14C-serotonin release assay (SRA) were sent for. Peripheral smear showed the absence of schistocytes. Given high clinical suspicion of HIT, the patient was started on intravenous argatroban infusion at the rate of 1 mcg/kg/hr, which was later increased to 1.5 mcg/kg/hr. However, over the course of 6 days, the platelet count failed to show any significant improvement. It only increased from a nadir of 20×109/L to 29×109/L after 6 days of continuous argatroban therapy. At this point, prednisone 60 mg once daily was added, and platelet count barely increased from 29x109/L to 40x109/L after 5 days of therapy. In the meantime, the HIT antibody test came back positive and so was SRA. Platelet factor 4 antibody test resulted positive as well. At this point, a diagnosis of autoimmune HIT was made and the patient was started on intravenous immunoglobulins infusion at the rate of 1 g/kg/24 hours. The patient received a total of two doses of intravenous immunoglobulins. With the first dose, the platelet count improved from 40×109/L to 66×109/L. With the second dose, it further improved to 73×109/L. At this point, the patient was started on oral apixaban 5 mg twice daily to anticoagulate, given chronic persistent atrial fibrillation and now acute CVA diagnosis. The patient was discharged home and followed up thereafter in the hematology office. His platelet count improved to 116×109/L on two weeks of outpatient follow-up. Figure 3 is a graphical representation of the chronology of platelet count along with different treatment strategies utilized. 

## Discussion

HIT is characterized by a platelet count drop of more than 50% from the highest value, typically occurring 5-14 days after the start of prophylactic or therapeutic dose heparin [1]. HIT occurs in approximately 1 in 5000 hospitalized patients, with a large variability among patient populations; especially in post-surgical cases, incidence rates of 1% to 3% have been reported after cardiac surgery [2]. Patients who receive unfractionated heparin for 7 to 10 days are described to be at the highest risk [3]. Heparin-induced thrombocytopenia (HIT) often results in thromboembolic complications ranging from deep venous thrombosis (DVT), pulmonary embolism (PE), thromboembolic stroke, and sometimes multisystem thromboembolic phenomena [4]. Skin necrosis, adrenal hemorrhagic necrosis, or post-intravenous heparin bolus anaphylactoid reactions are other rare but serious complications [5]. Thrombosis often occurs near the time of 50% platelet count decrease but can occur 1-2 days before platelets drop to 50%. The thrombotic risk is reported as high as 30 times in patients with HIT than in the control population [6]. 

The diagnosis of HIT is made mainly on the basis of clinical criteria but confirmed whenever possible by other laboratory tests. The tests for HIT antibodies are either immunoassays, e.g., enzyme-linked immunosorbent assay (ELISA), or functional tests, e.g., 14C-serotonin release assay (SRA) [7,8]. SRA is considered the ‘gold standard’ for HIT diagnosis [9]. The reported sensitivity and specificity of SRA are up to 100% and 97%, respectively, for unfractionated heparin (UFH) and low molecular weight heparin (LMWH)-treated patients [10]. Even subcutaneous heparin for DVT prophylaxis and heparin flushes used during hemodialysis have been reported to cause HIT [1,3]. Even after the discontinuation of heparin (UFH or LMWH), the risk of thrombosis remains high for a few days to weeks, even after the platelet count has returned to a normal level [7]. 

HIT can be further classified into two main types, mainly based on the timing of platelet drop after exposure to UFH or LMWH. Type 1, also called rapid onset HIT, occurs when platelet count drops as early as day 1 of heparin treatment. It usually resolves spontaneously; however, it can re-occur if followed by re-exposure within 1 month. This situation is usually seen in inpatient settings and is mild. Type 2 HIT is more common and is an antibody-mediated reaction and usually manifests after 5-14 days of heparin exposure (since it is antibody-mediated and it takes time for the body to form antibodies). This results in a serious reaction leading to a hypercoagulable state. Spontaneous HIT is mediated by Platelet Factor-4 antibodies and can occur in the absence of preceding heparin exposure. Delayed-onset HIT, as the name signifies, refers to a platelet count drop beginning days to a few weeks after heparin exposure and is the result of antibodies that cause platelet activation independently of heparin, mimicking an autoimmune disease. Persistent HIT is an entity where a drop in platelet count persists for weeks or even months. Autoimmune HIT is a term recently coined to describe delayed HIT, which is persistent, platelet antibodies are detected even in the absence of heparin, and platelet count recovers only in response to IVIG therapy [8]. These antibodies strongly activate platelets even in the absence of heparin and these patients have unusually severe thrombocytopenia [9,10]. There is emerging literature on autoimmune HIT, describing these antibodies and in vitro activity. A recent study found that serum from patients with autoimmune HIT contains highly pathogenic and aggressive antibodies. These autoantibodies (IgGs) have the ability to approximate two PF-4 molecules (hence overcoming inherent repulsive cationic PF-4 molecules without the need for heparin, which serves as a charge neutralizer in HIT). These highly reactive PF4-IgG-PF4 complexes can recruit heparin-dependent antibodies into aggressive immune complexes which continue to activate platelets [11,12,13,14]. It is also proposed that this process is likely facilitated by non-heparin polyanions found within platelets, such as chondroitin sulfate 5 and polyphosphates, thereby causing full HIT pathogenesis without the presence of heparin, and the prothrombotic effects of the antibodies continue apace [15,16, 17]. 

Treatment of HIT involves discontinuation of heparin on a high index of clinical suspicion while awaiting SRA results. An alternative anticoagulant should be started when acute venous or arterial thrombosis occurs [12]. Danaparoid, lepirudin, and argatroban are some of the drugs that have been shown to be effective in HIT. Treatment should continue for at least 5 days or until thrombosis has resolved. Anticoagulant treatment should overlap with conventional warfarin therapy for a few days. Usually, warfarin is continued for a period of at least 6 months [2], Recently, direct oral anticoagulants (DOACs) seem to be an attractive option in the management of HIT as an alternative to vitamin K antagonists (VKA). Treatment and diagnosis of autoimmune HIT remain diagnostic and therapeutic challenges. So far, the literature supports the use of intravenous immunoglobulin replacement therapy at the rate of 1 g/kg/24 hours for a total of two doses and watching for response [18]. There are new and experimental approaches for interrupting HIT antibody-induced platelet activation, e.g., 2-O, 3-O desulfated heparin,8 Syk inhibitor 9 [17,18,19,20].

## Conclusions

As challenging as it is, diagnosing and managing HIT remains a very difficult task. Autoimmune HIT should be suspected when a patient doesn’t respond to conventional treatment of HIT. Administration of intravenous immunoglobulins for autoimmune HIT treatment is lifesaving; however, practice varies depending on the institution. The anticoagulation choice after platelets have shown enough recovery remains a topic of discussion; however, we surmise that DOACs are a safer choice and should be utilized in the absence of other contraindications.
